# Feasibility of a Novel 3D Ultrasound Imaging Technique for Intraoperative Margin Assessment during Tongue Cancer Surgery

**DOI:** 10.3390/curroncol31080330

**Published:** 2024-08-01

**Authors:** Fatemeh Makouei, Theresa Dahl Frehr, Tina Klitmøller Agander, Giedrius Lelkaitis, Mette Hyldig Dal, Mikkel Kaltoft, Lisa Orloff, Merry Sebelik, Morten Bo Søndergaard Svendsen, Irene Wessel, Tobias Todsen

**Affiliations:** 1 Department of Otorhinolaryngology, Head and Neck Surgery and Audiology, Rigshospitalet, Copenhagen University Hospital, 2100 Copenhagen, Denmark; 2 Institute of Clinical Medicine, Faculty of Health Sciences, Copenhagen University, 2200 Copenhagen, Denmark; 3 Department of Applied Mathematics and Computer Science, Technical University of Denmark, 2800 Kongens Lyngby, Denmark; 4 Department of Pathology, Rigshospitalet, Copenhagen University Hospital, 2100 Copenhagen, Denmark; 5 Department of Cardiology, Bispebjerg Hospital, 2400 Copenhagen, Denmark; 6 Department of Otolaryngology-Head and Neck Surgery, Stanford University, Palo Alto, CA 94304, USA; 7 Department of Otolaryngology-Head and Neck Surgery, Emory University, Atlanta, GA 30308, USA; 8 Department of Computer Science, University of Copenhagen, 2100 Copenhagen, Denmark; 9 Copenhagen Academy for Medical Education and Simulation, The Capital Region of Denmark, 2100 Copenhagen, Denmark

**Keywords:** oral cancer, tongue squamous cell carcinoma, 3D ultrasound, surgical margins

## Abstract

Squamous cell carcinoma (SCC) of the tongue is the most prevalent form of oral cavity cancer, with surgical intervention as the preferred method of treatment. Achieving negative or free resection margins of at least 5 mm is associated with improved local control and prolonged survival. Nonetheless, margins that are close (1–5 mm) or positive (less than 1 mm) are often observed in practice, especially for the deep margins. Ultrasound is a promising tool for assessing the depth of invasion, providing non-invasive, real-time imaging for accurate evaluation. We conducted a clinical trial using a novel portable 3D ultrasound imaging technique to assess ex vivo surgical margin assessment in the operating room. During the operation, resected surgical specimens underwent 3D ultrasound scanning. Four head and neck surgeons measured the surgical margins (deep, medial, and lateral) and tumor area on the 3D ultrasound volume. These results were then compared with the histopathology findings evaluated by two head and neck pathologists. Six patients diagnosed with tongue SCC (three T1 stage and three T2 stage) were enrolled for a consecutive cohort. The margin status was correctly categorized as free by 3D ultrasound in five cases, and one case with a “free” margin status was incorrectly categorized by 3D ultrasound as a “close” margin. The Pearson correlation between ultrasound and histopathology was 0.7 (*p* < 0.001), 0.6 (*p* < 0.001), and 0.3 (*p* < 0.05) for deep, medial, and lateral margin measurements, respectively. Bland–Altman analysis compared the mean difference and 95% limits of agreement (LOA) for deep margin measurement by 3D ultrasound and histopathology, with a mean difference of 0.7 mm (SD 1.15 mm). This clinical trial found that 3D ultrasound is accurate in deep margin measurements. The implementation of intraoperative 3D ultrasound imaging of surgical specimens may improve the number of free margins after tongue cancer treatment.

## 1. Introduction

Oral squamous cell carcinoma (SCC) is the eighth-most common cancer, with a survival rate of less than 60% for patients surpassing 5 years [1,2]. SCC of the tongue represents the most prevalent form of oral cavity cancer, with surgery as the primary treatment modality [2,3]. The main objective in surgical oncology is to achieve complete tumor removal, along with a margin of healthy tissue surrounding the tumor, ensuring the effective eradication of cancer. Simultaneously, preserving as much healthy tissue as possible during tongue cancer surgery is crucial to maintain the patient’s quality of life, especially concerning vital functions such as swallowing and speaking. Positive or inadequate surgical margins (margin ≤ 5 mm) raise the risk of local cancer recurrence and often require adjuvant treatments, such as repeated surgery or radiation therapy, with greater morbidity and higher failure rates. Frozen section biopsy is commonly used to assess intraoperative margins in head and neck carcinomas [4]. Here, tissue samples from each margin of the surgical area are taken and then sent as frozen sections for microscopic examination by a pathologist while the patient is still under general anesthesia. A limitation of the frozen section analysis is that only a few samples are examined [5], which may not represent the entire margin. A meta-analysis found that frozen section analysis had a low sensitivity for close margins and that its use did not lead to better local control [6]. Additionally, frozen section analysis has a high cost [7] and may extend anesthesia duration by 15–45 min, increasing the risk of complications for the patient. Margin status analysis has, therefore, been a focal point in numerous clinical trials, with estimates suggesting that the percentage of inadequate surgical margins could be in the range of 30–65% [5,8,9,10].

To improve surgical cancer treatment, intraoperative imaging techniques aimed at optimizing complete tumor resection are imperative. Tumor-specific fluorescence optical imaging is a promising new technique with high sensitivity to detect positive resection margins [11]. However, the sensitivity for detecting close margins in the deep resection planes is lower due to the limited tissue penetration depth of fluorescence optical imaging [12]. This is especially problematic in oral cancer surgery, as the deep margins are involved in 87% of resection specimens with involved margins [13]. Instead, conventional 3D imaging modalities have been investigated for margin evaluation in oral SCC [14], including magnetic resonance imaging (MRI) [15,16] and computed tomography (CT) [3,17]. However, these methods come with significant drawbacks: they are costly and can substantially increase the duration of the operation as the surgical specimen needs to be transported from the operating room to the radiology department. Instead, ultrasound is a portable, dynamic, and cost-efficient imaging modality that offers high-resolution visualization of superficial structures [18]. Ultrasound has been found to be superior to MRI and CT for assessing the depth of invasion (DOI) in tongue SCC [3,18,19]. However, the limitations of ultrasound include its user-dependency and generation of two-dimensional images compared to a 3D volume with cross-sectional imaging. Instead, 3D ultrasound imaging may offer some promising potential in obtaining volumetric ultrasound images of tumors and overcoming these challenges [20,21]. Currently, 3D ultrasound imaging is conducted by a 3D probe [22,23,24], which is limited in its field of view, or the use of electromagnetic [25,26,27] or optical tracker [28] systems, which are difficult to use in an operating theatre [29]. Instead, we have developed a sensorless and compact three-dimensional (3D) ex vivo ultrasound system (3Sonic, prototype) involving a mechanical arm and a gridded marker to calibrate recorded 2D B-mode images into a 3D ultrasound volume. In this clinical trial, we aim to explore the diagnostic accuracy of using this novel 3D ultrasound technique for analyzing surgical margins during surgery for tongue cancer.

## 2. Materials and Methods

We conducted a prospective clinical study at the Department of Otorhinolaryngology, Head and Neck Surgery & Audiology, Copenhagen University Hospital, Rigshospitalet, including patients who were due for surgical treatment of tongue SCC. Ethical approval was granted in the form of an exemption letter from the Committee on Biomedical Research Ethics of the Capital Region of Denmark (registration number: 21017915), and the study protocol was registered with clinicaltrials.gov under the trial number NCT05740774. The inclusion criteria were patients above 18 years old with biopsy-proven oral tongue SCC scheduled for surgical treatment, and verbal and written informed consent were obtained from the patients before enrollment. The exclusion criteria were patients who had prior surgery or radiotherapy treatment for oral cavity cancer, had expected tumor invasion to the bone, or were unable to understand verbal or written information.

### 2.1. Ex Vivo 3D Ultrasound Imaging

**Figure 1:**
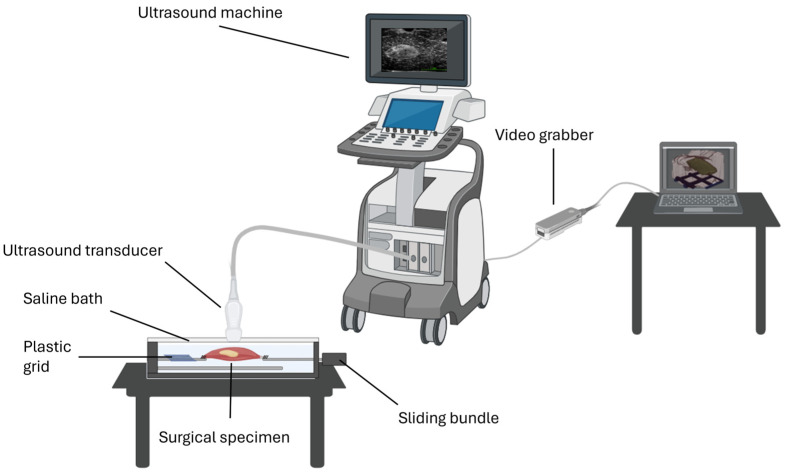
3Sonic setup—schematic of components of the 3D ultrasound imaging technique.

Under general anesthesia, the head and neck surgeon resected the tongue SCC using surgical magnifying glasses, with a safe margin of approximately 10 mm of healthy tissue around the tumor. The surgical margins of the resected specimen were clinically assessed by the surgeon and were fixated onto a piece of cork using needles. The specimen was marked either with a suture or by marking on the cork for orientation (anterior, posterior, medial, or lateral). Then, the surgeon took additional biopsies from the in vivo wound edges and sent them to the pathology department for frozen-section analysis. We then submerged the surgical specimen in saline solution for a 3D ultrasound scan at the operation theatre immediately after resection. We used a custom-made setup consisting of a motorized mechanical arm to move the ultrasound transducer above the surgical specimen, using saline as a coupling medium between the transducer and specimen. For calibration and to coordinate system retrieval, a plastic grid was fixated in the saline bath to provide the coordinate system information for the generation of a 3D ultrasound volume (see Figure 1).

Two different ultrasound machines were used in this study: the Hitachi Arietta 850 ultrasound system (Fujifilm, Ratingen, Germany), coupled with an L64 linear probe, and the BK 5000 (BK Ultrasound, Burlington, MA, USA), coupled with an L18 linear probe. Prior to the mechanical sweep of the probe, ultrasound image optimization was performed at the center position of the specimen. Volume construction and ultrasound data calibration were executed using a custom-made in-house script within the commercially available programming tool MATLAB 2022b (www.mathworks.com). Data were analyzed using MATLAB 2022b.

### 2.2. Three-Dimensional Ultrasound to Histopathology

**Figure 2:**
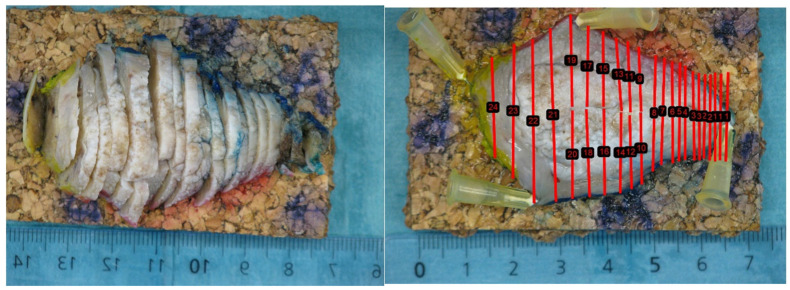
Parallel slicing of the surgical specimen by pathologist. The parallel red lines on the macro image represent the position of the pathology slices, and the numbers the order of which they were sliced.

After the surgery, the specimens were transferred to the pathology department for formalin fixation. Here, the samples were fixed in 10% formaldehyde solution for 24 h. The same pathologist (TKA) then performed parallel slicing at approximately equal intervals of 2 mm (See Figure 2).

**Figure 3:**
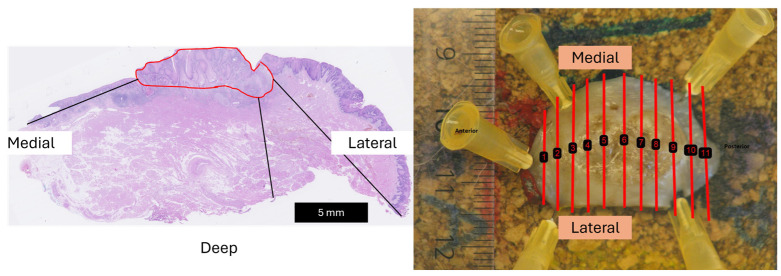
Schematic representation of the margin measurement directions on histopathology and macro image of the surgical specimen. The tumor region has been delineated in red on the histology image. The parallel red lines on the macro image represent the position of the pathology slices, and the numbers the order of which they were sliced.

The slices were transferred to cassettes for further processing and embedded in paraffin to form formalin-fixated paraffin-embedded (FFPE) blocks. From each block, a 4 μm slice was cut, mounted on glass slides, and stained with hematoxylin and eosin (H&E). Two experienced head and neck pathologists (TKA and GL) assessed the surgical margin status and delineated the tumor on the digital images of the histology slices. On each included patient, the pathologists measured the tumor area and the distance of the smallest deep, lateral, and medial margins of every slice (as illustrated in Figure 3). The measurements by the two pathologists were averaged and considered as the gold standard.

**Figure 4:**
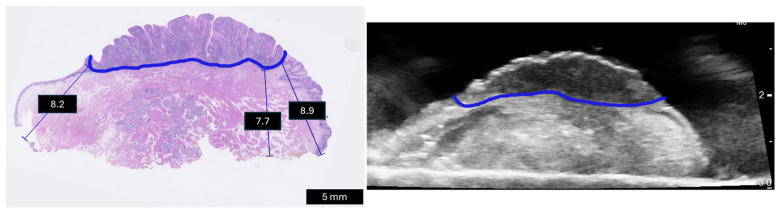
Pathology slice and its corresponding ultrasound frame. Tumor area is delineated by a pathologist and surgeon in blue on pathology and ultrasound, respectively. The pathologist measured the margins in mm.

Three-dimensional ultrasound images were correlated to the corresponding histopathology slices to evaluate the margin measurements. An example of the ultrasound-histology correlation is presented in Figure 4.

Four blinded head and neck surgeons manually segmented the tumor region and identified the slice-by-slice tumor area and surgical margins on the ultrasound images. We provided surgeons with a macro image of the surgical specimen to offer an overview of the sample. The selection of ultrasound frames was determined based on the specimen’s overall dimensions shown in the micro image and the markings by the pathologist on another image indicating the locations and orientations of the pathology slices. These chosen frames were compiled into a PowerPoint (PPT) file. Within this PPT file, surgeons were tasked with segmenting the tumor region and delineating the surgical margins on every slide. The surgeons conducted two rounds of measurements on the data.

To measure the tumor area and margins on ultrasound frames based on the drawings from the surgeons, we used a custom-made script in MATLAB 2022b. The results of the surgical margin assessment from the 3D ultrasound scan of the surgical specimen were compared to the final histopathology report.

### 2.3. Statistical Analysis

The histopathological margins were categorized as “free” (≥5 mm), “close” (1–5 mm), or “positive” (<1 mm). The smallest margin of all slices determined the final margin status for every patient. In this study, the positive class is defined as the close margins, meaning a true positive (TP) is when both pathology and ultrasound classify the margin as close. Likewise, the negative class refers to the free margins, meaning true negatives (TN) are patients where the margins are classified as free on both modalities. To evaluate the effectiveness of ultrasound in distinguishing between free and close surgical margins, we calculate its sensitivity and specificity. Sensitivity, or the true positive rate (TPR), is determined by dividing the number of true positives by the total number of positives. On the other hand, specificity, which is the true negative rate (TNR), is calculated as the ratio of true negatives to the total number of patients classified as negative. Likewise, the false positive rate (FPR) and the false negative rate (FNR) can be calculated.

For continuous variables, statistically significant differences were determined with an independent *t*-test. Modified Bland–Altman plots compared 3D ex vivo ultrasound measurements with histopathological as the reference standard. Paired-sample t-tests were performed to examine whether the mean differences between histological and US-based measurements were statistically significant. We defined the level of statistical significance at α = 0.05 with “ns” denoting non-significance. See Equations (A1) and (A2) in Appendix A for the mean and SD calculations.

## 3. Results

We prospectively enrolled eight patients diagnosed with SCC of the tongue and scheduled for primary surgical treatment between January 2023 and May 2023. We excluded two of the eight patients due to technical issues with the scanning, leaving us with six patients in the study. Of the six participants, three [50%] were women, and the mean age was 67 years [IQR, 9 years]). Three participants were clinically T-staged as T1, and three were T-staged as T2, all without suspicion of lymph node metastasis. Two of the six patients had free margins based on the final histology report, and four had close margins. One of the six participants had a cohesive invasion pattern (See Table 1). A total of 45 pathology slices with a tumor seen on histopathology were compared to the corresponding ultrasound images, with a median of 7.5 slices (range 3–13) for each patient.

The mean surgical margin from all slices from the different patients measured by the pathologists was 6.1 mm (IQR = 2.6 mm), 6.7 mm (IQR = 3.8 mm), and 8.6 mm (IQR = 4.0) for the deep, medial, and lateral margins, respectively. The mean absolute difference between the two pathologists’ measurements of the surgical margin from the slices was 0.5 mm (IQR = 0.5 mm), 1.4 mm (IQR = 1.4 mm), and 1 mm (IQR = 1.1 mm) for the deep, medial, and lateral margins, respectively. The Pearson correlation for their deep, medial, and lateral margin measurements were 0.9 (*p* = ns), 0.7 (*p* = ns), and 0.9 (*p* = ns), respectively. The maximum difference between the two pathologists’ measurements of the deep margin was 0.8 mm (patient 3), while the maximum difference was 2.3 mm (patient 1) and 1.9 mm (patient 4) for the medial and lateral margins, respectively.

**Figure 5:**
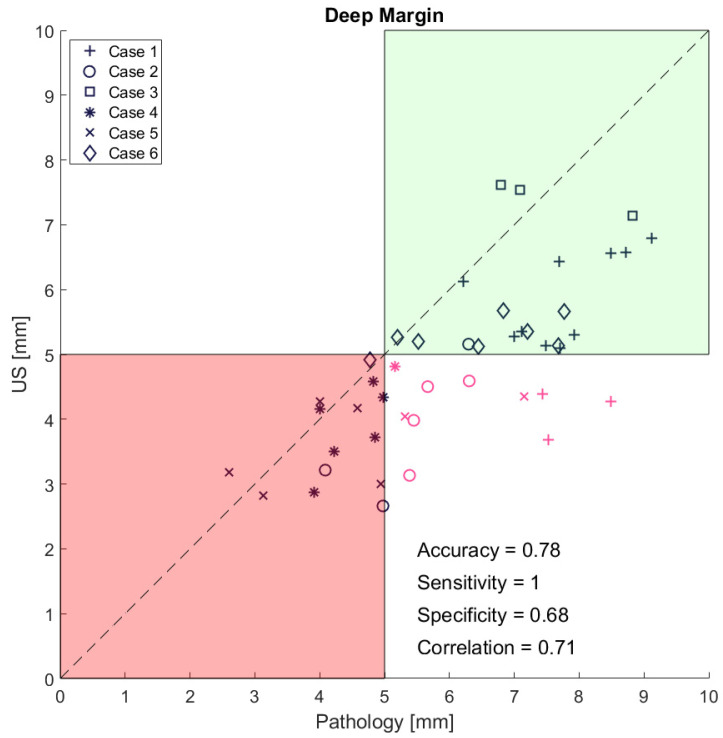
Ultrasound measurement vs. pathology for deep margin measurement for all histopathology slices from all patients. The red box in the left corner indicates inadequate or positive margins below 5 mm, and the green box in the right corner indicates safe margins above 5 mm. Measurements correctly classified by ultrasound are black, while the measurements misclassified by ultrasound to be below 5 mm are colored red.

The mean surgical margins from the ultrasound images (corresponding to the pathology slices) were 4.8 mm (IQR = 1.3 mm), 8.6 mm (IQR = 5 mm), and 7.3 mm (IQR = 1.8 mm) for the deep, medial, and lateral margins, respectively, measured as an average between the four head and neck surgeons. The median Pearson correlation for the four head and neck surgeons’ measurements of the deep, medial, and lateral margins on the ultrasound images was 0.7 (*p* < 0.01), 0.5 (ns), and 0.3 (*p* < 0.001), respectively. The mean differences between the measurements of the minimum surgical margins on the ex vivo ultrasound images and histopathology were 1.0 mm (SD 0.9 mm), 2.3 mm (SD 1.8 mm), and 1.8 mm (SD 1.7 mm) for the deep, medial, and lateral margins, respectively. The Pearson correlation between the ex vivo ultrasound images and histopathology measurements were 0.7 (*p* < 0.001), 0.6 (*p* < 0.001), and 0.3 (*p* < 0.05) for the deep, medial, and lateral margins, respectively. Considering all deep margins, the TPR, TNR, FPR, and FNR based on the comparison of all slices were 100%, 68%, 32%, and 0%, respectively (See Figure 5). By categorizing the margin status based on the minimum deep margin measurements by ultrasound, we found an accuracy of 83%, with one patient (case 1) incorrectly classified as close even though the deep margin was free on histopathology (Table 2). See Table A1 in Appendix A for more details about lateral and medial margins.

**Figure A1:**
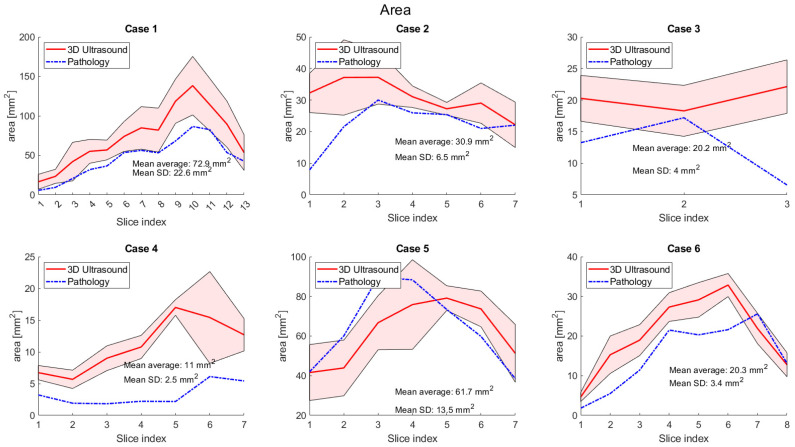
Slice-by-slice comparison of area measurements performed on histopathology and ultrasound respectively. The red shaded area indicates one standard deviation from the mean. The trend in ultrasound area measurements follows the pathology cancer regions in patients 1, 2, 5, and 6, while the correlation is less strong in patients 3 and 4.

**Figure A2:**
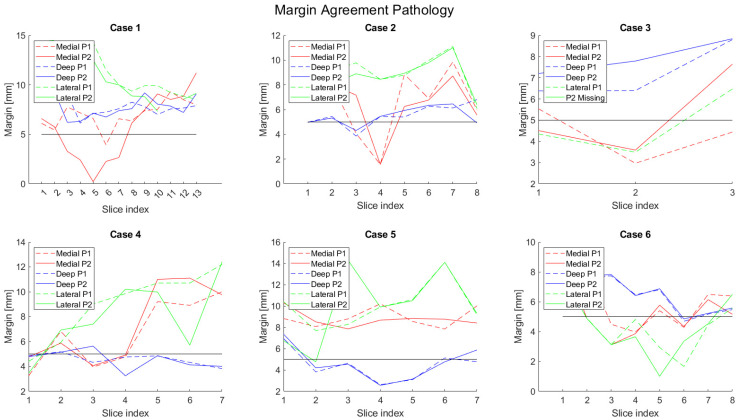
Agreement of lateral (green), deep (blue), and medial (red) margins between the two pathologists. The black line indicates the 5 mm margin threshold. P1 and P2 indicate the two pathologists.

**Figure A3:**
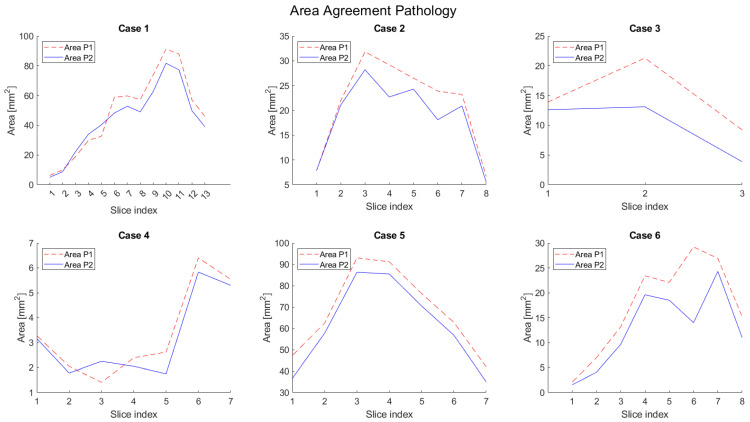
Agreement of tumor area measurements between the two pathologists.

The mean area from all slices from the different patients measured by the pathologists was 30.3 mm^2^ (IQR = 41.2 mm^2^). The mean absolute difference between the area of the slices measured by the two pathologists was 4.6 mm^2^ (IQR = 5.7 mm^2^). The Pearson correlation for their area measurements was 1.0 (*p* < 0.001). The maximum difference between the two pathologists’ measurements of the area was 15.2 mm^2^ (patient 6). The mean area measurement from the ultrasound images (corresponding to the pathology slices) was 41.5 mm^2^ (IQR = 39.8 mm^2^). See Figure A1 in Appendix A for more details about the tumor slice-by-slice area measurements on the ultrasound frames. The median Pearson correlation for the four head and neck surgeons’ measurements of the area was 0.9 (*p* < 0.01). The Pearson correlation between the ex vivo ultrasound images and histopathology measurements was 0.3 (*p* < 0.001). See Figure A2 and Figure A3 and Table A2 in Appendix A for more details about the area measurements from pathologists.

**Figure A4:**
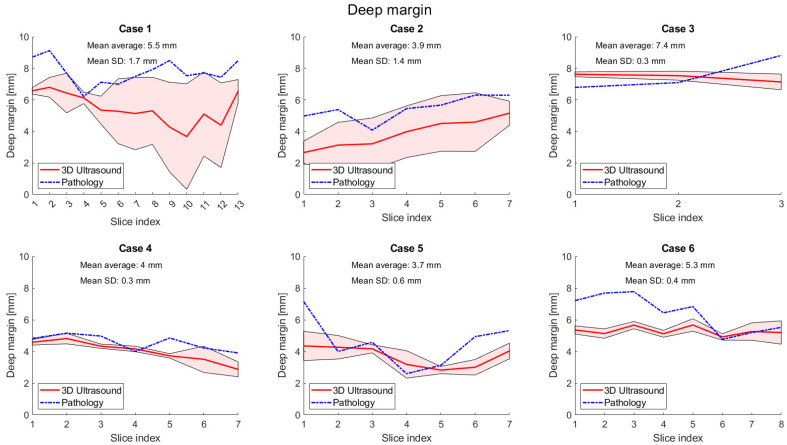
Slice-by-slice comparison of deep margin measurements performed on histopathology and 3D ultrasound. In these graphs, the dashed blue line represents the benchmark, which is the average of evaluations from two pathologists. The solid red line indicates the average result of 3D ultrasound assessments conducted by four head and neck surgeons. The shaded area shows the range of variability, or standard deviation, of the ultrasound measurements. The variation in ultrasound measurements depicted by the shaded region reflects the differences in tumor region delineations made by four head and neck surgeons analyzing 3D ultrasound data. Notably, in patients 1 and 2, a high standard deviation was observed. This variability suggests that the specific structure and tissue composition of the specimen can significantly impact the surgeons’ ability to consistently identify the tumor region.

The mean deep margin measurements averaged over all slices for patients 1–6 were 5.5 mm (SD 1.7 mm), 3.9 mm (SD 1.4 mm), 7.4 mm (SD 0.3 mm), 4 mm (SD 0.3 mm), 3.7 mm (SD 0.6 mm), and 5.3 mm (SD 0.4 mm), respectively (see Figure A4 in Appendix A). The measurements for patient 1 and patient 3 exhibited greater inaccuracies. The smallest normalized root mean square error (RMSE) was 0.1 (in patients 3 and 4), and the maximum error was 0.3 (in patients 1 and 2). See Table A3 and Equation (A3) in Appendix A for detailed information about the calculated RMSEs.

**Figure A5:**
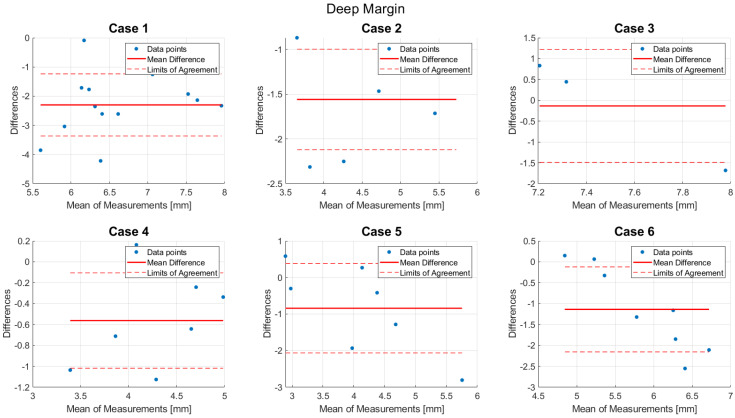
Bland–Altman depicting the difference between the measurement of ultrasound and histopathology on all six patients. The difference between the two is depicted by the solid red line. For case 1, it is −2.3 ± 1.1 mm; for case 2, it is −1.6 ± 0.6 mm; for case 3, it is −0.1 ± 1.3 mm; for case 4, it is −0.6 ± 0.5 mm; for case 5, it is −0.8 ± 1.22 mm; and for case 6, it is −1.1 ± 1.0 mm.

**Figure A6:**
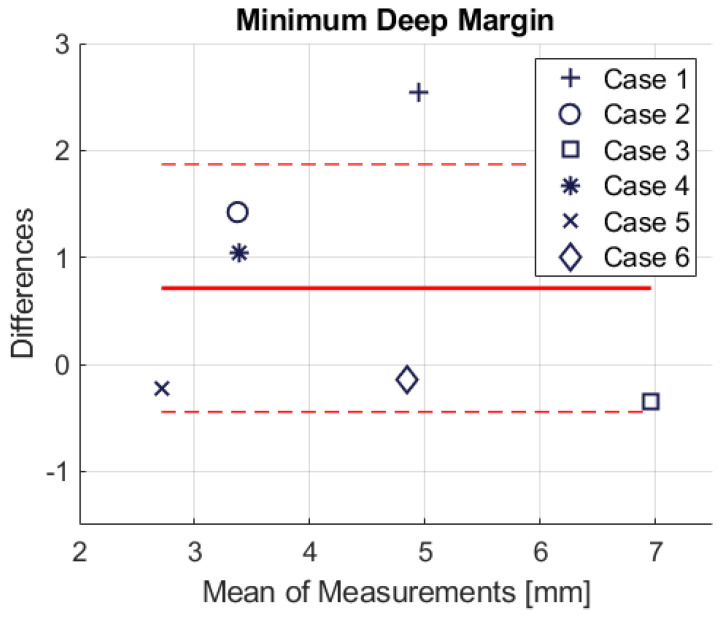
Bland–Altman plot of the difference between the smallest deep margin in ultrasound and the smallest deep margin in pathology. The solid red line depicts the mean difference between ultrasound and pathology, which is 0.7 mm. The dashed red line depicts the mean plus/minus the standard deviation of 1.15 mm.

The mean difference between the 3D ultrasound and histopathology in deep margin measurement was 0.7 mm (±SD 1.15 mm); see Figure A5 and Figure A6 in the Appendix A for more details.

**Figure A7:**
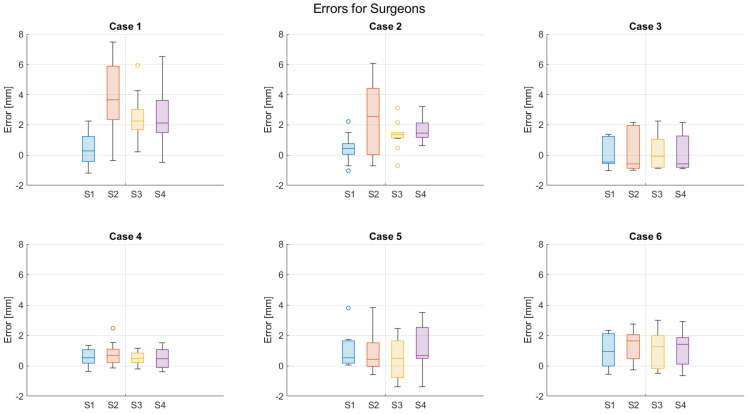
Error case-wise in mm for the deep margin for participating surgeons (S1–S4). Positive values mean ultrasound has underestimated the margin. Outliers are marked as circles outside the boxplots.

**Figure A8:**
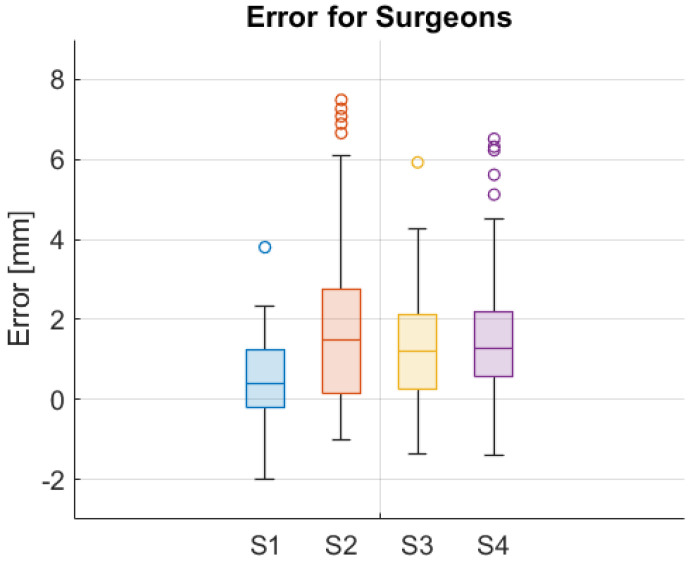
Error in mm for the deep margin for participating surgeons (S1–S4) for all patients. The median of the data points is marked as the line within the colored boxes, with their edges being the 75% and 25% percentile, respectively. The whiskers indicate the highest and lowest values, marking values outside these boundaries as outliers. Positive values mean ultrasound has underestimated the margin.

The median error for the surgeons were 0.4 mm (SD 1.0 mm), 1.5 mm (SD 2.2 mm), 1.2 mm (SD 1.3 mm), and 1.3 mm (SD 1.6 mm) (see Figure A7 and Figure A8 in the Appendix A for more details).

## 4. Discussion

This clinical feasibility trial evaluated the accuracy of a novel 3D ultrasound technique for ex vivo imaging of resected tongue SCC margins in the operating room. We found that 3D ultrasound was well correlated with histopathology, especially the deep margins, and could predict the final margin status in five out of six surgical tongue cancer cases. Three-dimensional ultrasound imaging is a promising modality that may be used intraoperatively to improve the rate of free margins after tongue cancer treatment.

Our trial is the first to explore a novel sensorless 3D ultrasound technique for the ex vivo imaging of surgical specimen margins during tongue cancer surgery. Compared to other 3D imaging modalities, the technique offers a low-cost image modality that could be performed with any commercial ultrasound machine in the operating room. A strength of the trial is that the patients were prospectively enrolled, and the surgical specimens were cut with thin “loaf of bread” slices that allowed us to correlate histopathology slices with corresponding ultrasound images. To establish gold standard pathology outcomes, two pathologists outlined the tumor area and measured three margins (lateral, medial, and deep) on each digital histology image for every slice. Further, we used four blinded head and neck surgeons to draw tumor areas on the 3D ultrasound images to calculate the mean area and decrease the ultrasound interrater variability. Our trial also has limitations to consider. We only included six patients from the same center in the final analyses, and our results may not be directly generalizable to the clinical setting. However, as the six surgical specimens were sliced using the “loaf of bread” technique, we were able to compare a total of 45 digital histopathology slices with the corresponding ultrasound images. We therefore believe the sample size is sufficient to explore whether the novel 3D ultrasound technique is feasible. Another limitation is that, even though the specimens were cut with thin parallel “loaf of bread” slices, the pathologist used a free-hand knife for cutting. Therefore, we cannot be sure all slices are evenly spaced, and only one histology slice from each FFPE is produced for the pathologists to evaluate. As a result, the ultrasound frames are not precisely aligned with the histopathology slices. It was, therefore, difficult to discriminate whether a low tumor area correlation between the histopathology and ultrasound images was due to unclear tumor borders on the ultrasound images or a misalignment of the corresponding ultrasound frames and histology slices. To address this issue, a possible solution could be using a slicing device to ensure more uniformly perpendicular and evenly spaced tissue slicing [30]. This could help improve the alignment between ultrasound images and histopathology slices, thereby enhancing the accuracy of tumor correlation. Further, the effect of tissue shrinkage due to the use of formalin and paraffin in tissue processing cannot be overlooked. El-Fol et al. have indicated that shrinkage could amount to up to 42% [31]. These factors could account for any discrepancies in findings between 3D US and histopathology.

In addition to the applications of ultrasound in our study, it is important to consider its role in the preoperative assessment of margins in tongue squamous cell carcinoma. For instance, Limongelli et al. demonstrated that high-definition ultrasound, when used for the evaluation of size and depth of early stage (cT1–T2) tongue squamous cell carcinoma, significantly aids in precise surgical planning. This approach, combined with three-dimensional diode laser surgery, not only enhances the accuracy of tumor excision but also improves patient outcomes by minimizing post-surgical complications and ensuring a higher rate of disease-free survival [32]. Our findings are aligned with several earlier studies that demonstrated the advantages of using ultrasound to assess the surgical margins in tongue SCC [10,19,33,34]. One previous study used an electromagnet tracking system to construct 3D ultrasound volumes [27]. However, this 3D technique is time-consuming and requires setting up an electromagnet tracking system, which is difficult to use in an operating room with limited space. Further, they used a radiologist for tumor segmentation—which is not available in the operating room—and still only achieved an accuracy of 62.5% correct margin assessments. Their lower diagnostic accuracy may be explained by their use of a low-frequency curvilinear transducer providing the 3D volume with lower image resolution, which makes it difficult to assess the tumor borders. Instead, our trial used a simple sensorless 3D ultrasound system with a high-frequency linear transducer, providing better image quality and better assessment of tumor margins (83% versus 62.5%). Further, we used four head and neck surgeons to delineate the tumor area and assess the margins on 3D ultrasound images to increase validity and explore interrater variability. Our clinical trial therefore mimics the real-world use of a perioperative imaging technique evaluated by the surgeon, as a radiologist is unlikely to be available in the operating room. Our results showed that 3D ultrasound could predict the correct final margin status based on the minimum deep margin for five out of six patients (Table 2). The only incorrectly categorized patient was patient 1 (FP), where ultrasound underestimated the minimum deep margin compared to histopathology and categorized the margin status as “close”, while the true category was “free”. In our study, the measurements of the deep margin using 3D ultrasound were generally smaller than those obtained through gold-standard histopathology. This indicates that, when using ultrasound imaging, surgeons tended to overestimate the extent of the tumor region. This may be explained by non-tumorous tissues, such as muscle, dysplastic regions, and areas of inflammation, appearing—like tumor tissues—hypoechoic on ultrasound images. This may lead to the misinterpretation of the area of cancer invasion to be larger than seen in histopathology. Inflammation around the tumor after a biopsy can appear hypoechoic on ultrasound and may lead to an overestimation of the tumor. However, as the biopsies were performed weeks before surgery in this study, we believe the inflammation around the tumor will be limited in this study. As we used a traditional high-frequency linear transducer (18–22 MHz) for ex vivo ultrasound imaging, a matrix or ultra-high frequency ultrasound transducer may achieve greater resolution to differentiate between malignant tumors and benign tissue.

The greatest concordance between the two pathologists was observed in the measurements of the deep margins, while the discrepancies in measurements for the lateral and medial margins were larger. One reason for variation among individuals could be their differing methods of compensating for folded or incomplete slices during measurement. Additionally, adjustments made for tissue that is not straight but rather curved and folded are also likely to vary between individuals. The higher pathologist variability also emphasizes that the mucosa margins are more difficult to reproduce and may therefore also be more difficult to assess with ultrasound. The evaluation of the performance of the four head and neck surgeons involved in the study showed a 1.1 mm median difference between the best and the worst performers. This indicates the fact that it is possible to improve the results by training the operator. Although all four surgeons were experienced with clinical ultrasonography, none of them had experience with the ultrasound assessment of tumor margins in ex vivo specimens. As experience with this 3D ultrasound method grows, there may be a learning curve or an opportunity to enhance sensitivity by educating surgeons in optimal techniques for margin interpretation.

A higher correlation between ultrasound and histopathology was observed in the deep margin compared to the lateral margins. The lateral margin is typically not problematic, but the deep margin often poses challenges, and ultrasound proves to be more effective in this area. However, ultrasound may not be the optimal modality for evaluating the mucosa. For this purpose, optical techniques such as fluorescence and narrow-band imaging (NBI) may yield better results. Autofluorescence imaging is a technology that has shown a significant impact in improving the detection of oral SCC when used as an adjunct to conventional oral examination [35,36]. Additionally, it has the potential to enhance the accuracy of measuring lateral surgical margins, potentially leading to better surgical outcomes. NBI provides high resolution enhancement of the tissue abnormality through selective wavelength reflectance magnifying endoscopy [37]. As an optical technique, it could enhance the visualization of mucosal neoplastic changes, which may better improve the lateral margin measurements.

If successful, future surgeon-based trials could also lead to the development of AI-based 3D ultrasound interpretations, as AI is becoming increasingly recognized as a powerful tool in diagnostic imaging.

This clinical trial found that our novel technique could conduct 3D ultrasound in a fast and feasible way in the operating room during surgery, with promising results. The sensitivity of 3D ex vivo ultrasound for deep margins was 100% in this trial, meaning that ultrasound detected all the patients with inadequate margins. However, ultrasound tends to report smaller deep margins, and the specificity was 68%, as some patients with margin >5 mm were classified as inadequate on the ultrasound images. This indicates that if 3D ultrasound finds safe margins >5 mm, frozen section analyses may not be necessary. Instead, frozen section analyses could be saved for cases where ultrasound measures inadequate or positive margins, and the 3D ultrasound can guide the surgeon to decide where and how much additional tissue should be cut for the frozen section analyses. Further, the 3D ultrasound volume will also provide the surgeon with 3D spatial visualization of tumor spread, which may improve the assessment of resection margins compared to the small biopsies from frozen section analysis. However, the major limitation of our study is the small number of cases (six). This small sample size significantly limits our ability to draw definitive conclusions, although the preliminary data suggest a tendency that supports our thesis. It is important to note that this study serves as a pilot study to validate the applicability of the proposed method. Future clinical trials with a larger cohort are necessary to provide more statistical power and explore how 3D ultrasound will change the perioperative decisions of the oncological surgeon and final patient outcome.

## 5. Conclusions

This pilot study found that 3D ultrasound imaging of surgical specimens shows potential as a fast and feasible imaging modality in the operating room. Three-dimensional ultrasound imaging demonstrated a good correlation to histopathology of the deep surgical margins and predicted the final margin status in five out of six surgical tongue cancer cases. However, the correlation with the lateral margins was lower and more difficult to predict. While 3D ultrasound imaging is a promising modality that may be used intraoperatively to improve the rate of free margins after tongue cancer treatment, these preliminary findings are based on a small sample size. Further research with a larger cohort is necessary to validate these results and confirm the clinical utility and reliability of this method.

## Figures and Tables

**Table 1:** Patient characteristics. No. pathology slices refer to slices containing tumor, and the margin status is determined by whether a case contains a deep margin below 5 mm. The staging is presented as cTNM (clinical TNM) or pTNM (pathological TNM), indicating whether the stage was determined preoperatively or postoperatively.

Patient No.	Gender	Age	cTNM or pTNM	No. Pathology Slices	Invasion Pattern	Perineural Invasion	Pathology Margin Status	Ultrasound Machine
1	Female	64	T2N0M0	13	Non-cohesive	Yes	Free	BK5000 (BK Ultrasound, Burlington, MA, USA)
2	Female	73	T2N0M0	7	Non-cohesive	Yes	Close	Arietta 850 (Fujifilm, Ratingen, Germany)
3	Male	81	T1N0M0	3	Non-cohesive	No	Free	Arietta 850 (Fujifilm, Ratingen, Germany)
4	Female	68	T1N0M0	7	Cohesive	No	Close	Arietta 850 (Fujifilm, Ratingen, Germany)
5	Male	50	T2N1M0	7	Non-cohesive	Yes	Close	Arietta 850 (Fujifilm, Ratingen, Germany)
6	Male	56	T1N0M0	8	Non-cohesive	No	Close	Arietta 850 (Fujifilm, Ratingen, Germany)

**Table 2:** The smallest deep margins measured by four head and neck surgeons (A–D) on 3D ultrasound and two pathologists (A + B) on histopathology images for all patients. Ultrasound and pathology status are based on the deep margin measurements. Abbreviations: FP = false positive, TP = true positive, TN = true negative.

Patient No.	Pathologist Minimum Deep [mm]				3D Ultrasound Minimum Deep [mm]						Margin Status		
	A	B	Difference	Mean	A	B	C	D	Mean	Mean Abs Error	3D US	HP	Diagnosis
1	6.1	6.2	0.1	0.7	5.9	0.4	3.8	1.8	3.0	2.3	Close	Free	FP
2	3.9	4.3	0.5	0.4	3.1	1.9	2.7	2.4	2.5	2.2	Close	Close	TP
3	6.4	7.2	0.8	0.8	7.5	6.7	7.2	7.1	7.1	2.4	Free	Free	TN
4	3.8	3.3	0.6	0.5	2.8	2.6	3.2	2.6	2.8	2.0	Close	Close	TP
5	2.6	2.6	0.1	0.4	2.7	2.8	3.1	2.3	2.6	2.1	Close	Close	TP
6	4.7	4.9	0.2	0.1	5.1	4.4	4.9	4.8	4.8	0.2	Close	Close	TP
Overall	4.6	4.8	0.4	0.5	4.5	3.1	4.2	3.5	3.8	1.9	-	-	-

## Data Availability

The data presented in this study are available in this article.

## Appendix A

The mean of the results was calculated as follows:

(A1) $$\mu \left(k\right)=\frac{1}{n}\frac{1}{m}\sum_{i=1}^{n}\sum_{j=1}^{m}u_{ij}\left(k\right),$$

where *k* is the slide number, *n* is the number of repeated drawings on the same slide, *m* is the number of physicians, and $u_{ij}$ is the ultrasound measurement, whether it be deep margin or area. The standard deviation (SD) was calculated similarly, as follows:

(A2) $$SD(k)=\sqrt{\frac{1}{nm-1}\sum_{i=1}^{n}\sum_{j=1}^{m}{\left(u_{ij}(k)-\mu (k)\right)}^{2}}.$$

For comparison with the gold-standard histopathology measurements, the RMSE is reported, calculated with the following equation:

(A3) $$RMSE=\sqrt{\frac{1}{K}\sum_{k=1}^{K}{\left(\mu \left(k\right)-h\left(k\right)\right)}^{2}}$$

where *K* is the total number of slides, and *h*(*k*) is the gold-standard histopathology measurement.

**Table A1:** The smallest lateral and medial margins measured by both 3D ultrasound (3D US) and histopathology (HP) for all patients. The error is listed with positive numbers meaning ultrasound underestimated the margins, and negative numbers meaning ultrasound overestimated the margins. The Pearson correlation is computed using all measurements of a margin from a patient, and likewise, a *p*-value is computed using a paired sample *t*-test with “ns” denoting non-significance.

Patient No.	Minimum Medial [mm]				Minimum Lateral [mm]			
	3D US	HP	Error	Pearson (pval)	3D US	HP	Error	Pearson (pval)
1	6.6	8.6	2.0	−0.1 (<0.01)	6.3	3.1	−3.2	0.7 (<0.001)
2	4.9	6.2	1.4	0.9 (<0.001)	6.4	1.6	−4.8	0.5 (<0.01)
3	5.1	3.5	−1.6	0.1 (ns)	3.8	3.3	−0.5	−0.5 (ns)
4	4.9	3.9	−1.0	0.9 (ns)	5.8	4.0	−1.8	0.9 (ns)
5	6.3	6.1	−0.1	−0.6 (<0.05)	11.7	8.3	−3.4	0.1 (<0.001)
6	6.9	2.0	−5.0	0.6 (<0.001)	4.0	3.8	−0.2	0.6 (ns)

**Table A2:** A table of the mean absolute difference between the two pathologists’ measurements. The most consistent measurements between the two pathologists were observed for the deep margins, indicating the highest level of agreement in this area. Conversely, the greatest discrepancy in their assessments was noted for the medial margins. On average, the smallest margin difference was ~0.5 mm for the deep margins, while the largest average difference was 1.3 mm for the medial margins.

Mean Absolute Difference [mm]	Case 1	Case 2	Case 3	Case 4	Case 5	Case 6	Average over Cases
Medial	2.2	1.0	1.6	1.0	1.0	0.9	1.3
Deep	0.7	0.4	0.7	0.5	0.4	0.1	0.5
Lateral	0.9	0.3	0.2	1.4	1.8	0.7	0.9
Area	6.8	2.8	5.0	0.5	6.7	4.5	4.4

**Table A3:** RMSE [mm^2^] comparing fresh ultrasound specimens to histopathology case-wise. RMSE values have been normalized by the largest area/deep margin measured by histopathology for each case.

Case	1	2	3	4	5	6
Deep	0.3	0.3	0.1	0.1	0.2	0.2
Medial	0.3	0.4	0.2	0.2	0.4	0.2
Lateral	0.2	0.3	0.2	0.1	0.3	0.5
Area	0.3	0.4	0.6	1.3	0.2	0.3
